# Psychosocial and Demographic Factors That Influence HbA1c Testing Frequency in Diabetics: Data From the 2018 Behavioral and Risk Factor Surveillance Survey (BRFSS)

**DOI:** 10.7759/cureus.29798

**Published:** 2022-09-30

**Authors:** Swarada Yadav, Ashish Sarangi, Wail Amor, Kenneth Nugent

**Affiliations:** 1 Internal Medicine, University of Alabama at Birmingham, Birmingham, USA; 2 Psychiatry and Behavioral Science Education, University of Missouri School of Medicine, Columbia, USA; 3 Psychiatry, Texas Tech University Health Sciences Center, Lubbock, USA; 4 Internal Medicine/Pulmonary and Critical Care Medicine, Texas Tech University Health Sciences Center, Lubbock, USA

**Keywords:** diabetic knowledge, diabetic care, diabetes, psychosocial, hba1c

## Abstract

Background

HbA1c testing is an essential measure of glycemic control in diabetic patients. This study aims to determine the social and psychological determinants that have a role in the frequency of HbA1c testing in diabetics.

Methods

We used data from the Behavior and Risk Factor Surveillance Survey to develop a hierarchical linear regression model to examine associations between annual HbA1c testing frequency and the following types of variables: demographics, socioeconomic factors (SES), living environment, healthcare access, psychosocial factors, clinical factors, and diabetes self-care and knowledge.

Results

The study included 18,505 diabetics from the BRFSS 2018 dataset with a mean age of 61.2 years. There were significant associations between age, gender, race, insurance status, SES, healthcare access, psychosocial factors, and HbA1c testing frequency. Being American Indian or Alaskan Native was associated with increased HbA1c testing frequency compared to Whites, and non-Hispanics. Higher education/income was associated with increased HbA1c testing frequency. Regular doctor visits for diabetes were associated with increased HbA1c testing frequency.

Conclusions

In our analysis of a national survey, income, education level, and diabetes-specific care variables were significantly associated with the frequency of HbA1c testing. These results help identify patient groups that need more attention in managing diabetes, including the use of HbA1c testing.

## Introduction

Glycosylated hemoglobin (HbA1c) is an essential indicator of glycemic control in the management of diabetes [1]. The American Diabetes Association (ADA) has recommended HbA1c as a potential substitute for fasting blood glucose for monitoring and diagnosing diabetes [2,3]. Currently, HbA1c testing is recommended twice annually in patients with controlled diabetes, and four times a year in patients with uncontrolled diabetes [4]. HbA1c testing is convenient and easier for patients since it does not require fasting and requires only a single blood sample [5]. HbA1c highly correlates with diabetes-related complications, is highly standardized and accurate, has low intra-individual variability, and is unaffected by short-term lifestyle changes [6,7]. Several studies have reported that individuals who get tested at three months are more likely to achieve good glycemic control [8]. In addition, one of the goals of Healthy People 2020, a national program to improve the health of Americans across a 10-year span, has increased HbA1c testing frequency to twice annually in 72.9% of diabetics as one of its goals [9]. To promote and increase the use of HbA1c testing by diabetics, it is crucial to assess factors that may affect the testing frequency in these individuals [10].

Several studies have reported that demographic factors, psychosocial factors, and factors related to healthcare access and utilization have important implications for diabetes management. Psychosocial factors connect individual behaviors and social surroundings and an illness and may have an essential role in the long-term effects of diabetes in these patients [11]. The ADA suggests that achieving optimal glycemic control is possible in pre-diabetics and diabetics if they have social support, access to healthcare, and help in developing self-management skills [12]. Furthermore, with increasing evidence that HbA1c is not only a predictor of diabetic status but is also a risk factor in the development of other chronic illnesses, such as cardiovascular diseases and cancer, it is imperative to evaluate the association between the above factors and frequency of HbA1c testing [13]. Given the lack of comprehensive studies in these areas, this study analyzed several demographic and psychosocial factors and their association with the frequency of HbA1c testing, to identify future interventions and care for people with diabetes and help them achieve better outcomes in the management of their disease. Psychological factors such as comorbid depression and anxiety can significantly impact the frequency of A1c testing and overall health-seeking behaviors in patients with diabetes.

## Materials and methods

Data for this study were from self-reported responses to the questionnaire of the 2018 Behavioral and Risk Factor Surveillance Survey (BRFSS). The BRFSS is an annual national longitudinal telephone survey that collects data on risk behaviors related to health, chronic health conditions, and the use of preventive services. The study in 2018 had a sample size of 136,967. Our analytic sample included all the individuals who responded ‘yes’ to the survey question ‘Ever told you have diabetes? (N = 20,481). Individual respondents with missing values in any of the variables that were used in this study were excluded (N = 1,976). Our outcome variable was determined using the question ‘About how many times in the past 12 months has a doctor, nurse, or other health professional checked you for A1c?’ which was recorded as a continuous variable and was retained as a continuous variable.

Variables of interest

Variables of interest associated with HbA1c testing included a series from the following categories.

Demographic Covariates

This category includes age, sex, race/ethnicity, and marital status. Age was analyzed as a continuous variable. We used the imputed race/ethnicity BRFSS 2018 variable, and included the following categories: White, Non-Hispanic; Black, Non-Hispanic; Asian, Non-Hispanic; American Indian/Alaskan Native, Non-Hispanic; Hispanic; and other races. Marital Status was collapsed as: (1) married; (2) divorced, widowed, separated; (3) never married.

Socioeconomic Status

Socioeconomic status variables included income, education level, and employment status. Income was determined using self-report to the question ‘Is your annual household income from all sources treated as a continuous variable? Responses ranged from $10,000- $150,000. Employment status in BRFSS consisted of the following responses ‘Employed for wages’, ‘Self-employed’, ‘Out of work for 1 year or more, ‘Out of work for less than 1 year’, ‘A homemaker’, ‘A student’, ‘Retired’, and ‘Unable to work,’ which was collapsed to: ‘Employed’ or ‘unemployed’ for the analysis. Educational status was assessed using responses for the question ‘What is the highest grade or year of school you completed?’ which was collapsed into ordinal variables with categories such as ‘Never attended school’, ‘Grade 1 - High School Diploma or GED’, ‘College graduate’ and above.

Living Environment

The living environment of the study participant was evaluated using variables such as ‘Metropolitan status’ which had responses ‘metropolitan counties’, and ‘non-Metropolitan counties. Similarly, the urban vs rural living of the study participants was determined. Additionally, to determine if the participants lived in a rented or owned apartment or house the responses to the question ‘Do you own or rent your home?’ which included ‘Own’, ‘rent’, ‘other arrangements’, ‘don’t know/not sure, ‘refused’ were used. However, the variable was collapsed to include only the responses ‘rent’, and ‘own’.

Healthcare Access and Utilization

Healthcare access and utilization among the participants were evaluated using the questionnaires ‘Do you have one person you think of as your personal doctor or health care provider?’, ‘About how long has it been since you last visited a doctor for a routine checkup?’, ‘Was there a time in the past 12 months when you needed to see a doctor but could not because of cost?’ in the BRFSS.

Psychosocial Factors

Questions such as ‘(Ever told) you have a depressive disorder (including depression, major depression, dysthymia, or minor depression)?’, ‘Now, thinking about your mental health, which includes stress, depression, and problems with emotions, for how many days during the past 30 days was your mental health not good? (Which was recorded as a continuous variable however, we broke this up into categorical variables with individuals reporting poor mental health <14 days b. poor mental health > 14 days) to determine the psychological status of individuals suffering from diabetes. Furthermore, the functional independence of individuals was assessed using the question ‘Because of a physical, mental, or emotional condition, do you have difficulties doing errands alone such as visiting a doctor's office or shopping?’.

Clinical Factors

Clinical factors such as insulin use and comorbidity were determined using the questionnaire ‘Are you now taking insulin?’ which had a yes or no response. Comorbidity was determined using a series of questions related to the presence or absence of chronic disorders. They were determined using questions such as ‘(Ever told) you have chronic obstructive pulmonary disease, C.O.P.D., emphysema or chronic bronchitis?’, ‘(Ever told) you had a stroke, ‘(Ever told) you had angina or coronary heart disease?’, ‘(Ever told) you have kidney disease? (Do NOT include kidney stones, bladder infection, or incontinence.)’, and ‘(Ever told) you had asthma?’ all of which were yes or no responses. Based on the number of yes responses, comorbidity count was calculated and treated as a continuous variable.

Diabetes Self-Care and Knowledge

The study participants’ level of knowledge and self-care in diabetes was determined using the following survey questions: ‘Have you ever taken a course or class on how to manage your diabetes yourself?’, ‘About how many times in the past 12 months have you seen a doctor, nurse, or another health professional for your diabetes?’, ‘About how many times in the past 12 months has a health professional checked your feet for any sores or irritations?’ (which was recorded as a continuous variable, later collapsed into a dichotomous variable with yes or no response for the ease of analysis)’, ‘Adults who reported doing physical activity or exercise during the past 30 days other than their regular job’ (which was a calculated variable for adults on physical activity based on the participants' response on physical activity if exercise in the past month excluding their regular job). ‘About how often do you check your blood for glucose or sugar?’ (which was recorded as a continuous variable, later collapsed into a dichotomous variable with yes or no response for the ease of analysis)’,’ How old were you when you were told you have diabetes?’ to determine the duration of diabetes and finally, ‘Has a doctor ever told you that diabetes has affected your eyes or that you had retinopathy?’.

Statistical analyses

The data were analyzed in three parts: first, means and frequencies including standard error (SE) of the variables to describe the study population; second, Pearson correlation to test the association between the frequency of HbA1c testing and demographics, psychosocial, socioeconomic, determinants of health variables, and knowledge of self-care; third, a hierarchical multiple linear regression model to evaluate the association between the variables and HbA1c testing, with variables entered in blocks. Model 1 tested demographic factors, Model 2 added socioeconomic factors (SES) variables, Model 3 added living environment, Model 4 added health care access and utilization, Model 5 added psychosocial variables, Model 6 added clinical factors, and last Model 7 added diabetes self-care and knowledge variables. All these statistical analyses were performed using SAS (version 9.4) and analyzed using the appropriate survey methodology. A two-tailed alpha of 0.05 was assessed for significance.

## Results

Baseline demographic characteristics of our diabetic sample (n=18,505) are reported in Table 1. Our sample was 51.3% female and 48.7% male, with a racial/ethnic distribution of 62.2% non-Hispanic White, 17.8% non-Hispanic Black, and 14.4% Hispanic. Over half (53%) of the sample never attended school or obtained a GED, 24.4% reported an annual income <$10,000, and approximately 50% reported an annual income over $25,000. Nearly all of the participants (91%) had health insurance (Table 1).

**Table 1:** Demographic characteristics of the sample (N = 18,505) *p < 0.05, **p < 0.01, ***p < 0.0001

	Percentage or Mean (Standard error)
Age (years)	61.2 (0.22)
Gender	
Male	48.7 (0.87)
Female	51.3 (0.87)
Race/Ethnicity	
White, Non - Hispanic	62.2 (0.88)
Black, Non - Hispanic	17.8 (0.58)
Asian, Non - Hispanic	2 (0.31)
American Indian, Alaskan native, Non – Hispanic	2 (0.21)
Hispanic	14.4 (0.92)
Other Race, Non - Hispanic	1.7 (0.16)
Marital Status	
Married	53.1 (0.88)
Separated/Divorced	18.0 (0.63)
Widowed	14.5 (0.53)
Never Married/Not Married	14.4 (0.63)
Annual Income	
	24.4 (0.85)
$10,000 - $14,999	7.8 (0.59)
$15,000 - $19,999	9.4 (0.45)
$20,000 - $24,999	10.3 (0.55)
$25,000 - $34,999	10.0 (0.48)
$35,000 - $49,999	11.4 (0.50)
$50,000 - $74,999	9.70 (0.44)
$75,000 +	16.9 (0.59)
Insurance	
Insured	91.0 (0.53)
Uninsured	9.0 (0.53)
Education	
Never attended school Grade 1 - high School graduate/GED	0.85 (0.14) 52.6 (0.84)
College Graduate and above	46.6 (0.84)

HbA1c testing frequency and predictors

We evaluated the correlation between demographic, social determinants of health, knowledge, and self-care in diabetes variables and annual HbA1c testing frequency as our outcome (Table 2).

**Table 2:** Pearson’s correlation for association between the frequency of HbA1c and demographic, social determinants of health variables, knowledge and self-care in diabetes. *p < 0.05, **p < 0.01, ***p < 0.0001

	Correlation Coefficient
Age (years)	-0.006
Gender	0.02**
Race/Ethnicity	-0.01
Marital status	0.002
Employment Status	0.05***
Insurance	0.07***
Socioeconomic Factors	
Income	0.02*
Education	0.04***
Employment Status	0.05***
Living Environment	
Metropolitan status	0.004
Urban vs Rural	-0.002
House	-0.01
Healthcare access / Utilization	
Personal Doctor	-0.09***
Routine Checkups (for other than Diabetes)	-0.1***
No access to doctor due to cost	0.03***
Psychosocial Factors	
Poor Mental Health	0.05***
Diagnosed Depression	-0.04***
Functional Independence	-0.05***
Clinical Factors	
Insulin use	-0.2***
Comorbidity	0.08***
Diabetic Knowledge/Self-care	
Diabetic Education	-0.13***
Doctor visits for Diabetes	0.2***
Regular Feet check	0.1***
Eye affected due to Diabetes	-0.09***
Physical Exercise	0.008
Blood Sugar Monitoring	-0.2***

Gender, insurance, SES, poor mental health, and comorbidity were positively associated with HbA1c testing frequency; factors listed under healthcare access and utilization (except for no access to the doctor due to cost), psychosocial factors (except poor mental health), clinical factors, diabetic knowledge, the diabetic eye, blood sugar monitoring were all negatively associated with frequency.

Hierarchical linear regression

Hierarchical regression was used to analyze the association between the variables of interest in HbA1c testing frequency (Table 3).

**Table 3:** Hierarchical regression models for influence of social determinants, clinical factors, psychosocial, healthcare access and utilization and self-care on the frequency of testing for glycosylated hemoglobin (HbA1C). *p < 0.05, **p < 0.01, ***p < 0.0001

	Model 1 (Demographic)	Model 2 (SES)	Model 3 (Living Environment)	Model 4 (Healthcare Access and Utilization)	Model 5 (Psychosocial)	Model 6 (Clinical Factors)	Model 7 (Diabetes knowledge and self-care)
Age (yrs.)	-0.003 (-0.006 , 0.0002)	-0.007*** (-0.01,-0.004)	-0.007*** (-0.01,-0.004)	-0.01*** (-0.01, -0.007)	-0.007*** (-0.01, -0.003)	-0.01*** (-0.01, -0.007)	-0.008* (-0.01, -0.002)
Gender							
Male (Ref = Female)	-0.09** (-0.16 , -0.03)	-0.09** (-0.14, -0.02)	-0.09** (-0.15, -0.02)	-0.07*** (-0.13, -0.006)	-0.05 (-0.11, 0.01)	-0.1* (-0.2, -0.02)	-0.1 (-0.2, -0.02)
Race/Ethnicity							
White/Non-Hispanic (ref)	-	-	-	-	-	-	-
Black/Non-Hispanic	0.07 (-0.002 , 0.16)	0.09	0.09 (0.01, 0.17)	0.08 (-0.003, 0.15)	0.08 (-0.007, 0.17)	0.1* (0.009, 0.3)	0.04 (-0.1. 0.2)
Asian/Non-Hispanic	0.02 (-0.31 , 0.35)	0.04	0.04 (-0.29, 0.37)	0.06 (-0.27, 0.39)	0.09 (-0.24, 0.41)	0.3 (-0.5, 0.98)	0.2 (-0.5, 0.9)
American Indian, Alaskan native	0.3*** (0.2 , 0.5)	0.3*** (0.2 , 0.5)	0.3*** (0.2 , 0.5)	0.4*** (0.3, 0.6)	0.4** (0.3, 0.6)	0.5* (0.2, 0.7)	0.4* (0.1, 0.7)
Hispanic	-0.3*** (-0.5 , -0.2)	-0.3*** (-0.4 , -0.1)	-0.3*** (-0.4 , -0.1)	-0.2** (-0.4, -0.1)	-0.2* (-0.4, -0.1)	-0.1 (-0.4, 0.1)	-0.1 (-0.4, 0.1)
Other Race	0.09 (-0.09,0.27)	0.1 (-0.11,0.3)	0.08 (-0.1, 0.3)	0.1 (-0.07, .03)	0.1 (-0.08, 0.3)	-0.08 (-0.37, 0.2)	-0.09 (-0.36, 0.2)
Insurance yes (no is ref)	-0.6*** (-0.7,-0.4)	-0.5*** (-0.7,-0.4)	-0.5*** (-0.7,-0.4)	-0.3*** (-0.5, -0.2)	-0.3*** (-0.5, -0.2)	-0.4* (-0.6, 0.1)	-0.3* (-0.5, -0.02)
Socio-Economic Factors							
Income		0.02** (0.007, 0.03)	0.02** (0.006, 0.03)	0.01* (0.0004, 0.03)	0.01* (0.006, 0.03)	0.02 (-0.001, 0.04)	0.02* (0.001,0.04)
Education							
Never attended school		-0.6** (-0.99,-0.19)	-0.6** (-0.98,-0.18)	-0.5* (-0.9,-0.12)	-0.5* (-0.9,-0.09)	-0.2 (-0.9,0.6)	-0.03 (-0.8,0.7)
Grade 1 - High school graduate		-0.1*** (-0.2,-0.07)	-0.1*** (-0.2,-0.07)	-0.1*** (-0.2,-0.07)	-0.1*** (-0.2,-0.07)	-0.1** (-0.2,-0.04)	-0.08 (-0.2,0.02)
College Graduate and above		-	-	-	-	-	-
Employment Status		0.3*** (0.26, 0.42)	0.3*** (0.26, 0.42)	0.3*** (0.26, 0.42)	0.3*** (0.19, 0.34)	0.2** (0.1, 0.4)	0.2* (0.06, 0.4)
Living environment ^(a)^							
Metropolitan status			0.03 (-0.05, 0.11)	0.03 (-0.05, 0.12)	0.03 (-0.05. 0.11)	0.01 (-0.1, 0.1)	0.01 (-0.1, 0.14)
Urban vs rural			-0.04 (-0.14, 0.06)	-0.03 (-0.13, 0.07)	-0.03 (-0.13, 0.06)	-0.05 (-0.2, 0.1)	-0.07 (-0.2, 0.1)
Housing (rent vs own)			-0.02 (-0.06, 0.02)	-0.02 (-0.06, 0.03)	-0.02 (-0.06, 0.02)	-0.03 (-0.1, 0.03)	-0.01 (-0.08, 0.05)
Healthcare access ^(b)^							
Access to personal doctor				-0.5*** (-0.7, -0.4)	-0.5*** (-0.7, -0.4)	-0.4** (-0.6, -0.2)	-0.3* (-0.5, -0.05)
Routine checkups (other than diabetes)				-0.4*** (-0.5, -0.4)	-0.4*** (-0.5, -0.4)	-0.4*** (-0.6, -0.3)	-0.3*** (-0.5, -0.2)
No access to doctor due to cost				0.08 (-0.01, 0.19)	0.2** (0.05, 0.25)	0.1 (-0.03, 0.3)	0.1 (-0.1, 0.2)
Psychosocial factors ^(c)^							
Poor mental health					0.2*** (0.1, 0.3)	0.03 (-0.1, 0.2)	0.03 (-0.10, 0.2)
Diagnosed depression					-0.06 (-0.14, 0.12)	0.1 (-0.1, 0.13)	0.02 (-0.09, 0.1)
Functional independence					-0.2*** (-0.3, -0.1)	-0.1* (-0.3, -0.01)	-0.1 (-0.2, 0.02)
Clinical factors							
Insulin use							
No (ref)						-	-
Yes						-0.8*** (-0.9, -0.7)	-0.5*** (-0.6, -0.3)
Comorbidity^(e)^						0.2*** (0.1, 0.3)	0.2*** (0.1, 0.2)
Diabetes self-care and knowledge							
Duration of diabetes							-0.004 (-0.008, 0.00006)
Diabetic education							-0.3*** (-0.4, -0.2)
Doctor visits for diabetes							1.2*** (1.05, 1.33)
Regular feet check							0.5*** (0.4, 0.7)
Diabetic eye check							-0.09 (-0.21, 0.03)
Physical exercise							0.05 (-0.04, 0.2)
Blood sugar monitoring							-0.6*** (-0.8, -0.5)

In Model 1 accounting for demographics only, compared to females, the male gender was associated with a 0.09 unit decrease in the frequency of HbA1c testing [-0.09 (-0.16, -0.013)]. Similar observations were made in all models except Models 5 and 7, which considered variables related to psychosocial factors and factors related to diabetes knowledge and self-care, respectively. Compared to non-Hispanic Whites, American Indian and Alaskan Native ethnicity was associated with a 0.3 unit increase in the frequency of HbA1c testing [0.3 (0.2, 0.5)]; Hispanic ethnicity was associated with a 0.3 decrease in the frequency of HbA1c testing [-0.3 (-0.4, -0.1)] after accounting for demographic factors, SES, and living environment. Compared to not having insurance, having insurance was associated with a 0.6 unit decrease in HbA1c testing frequency [-0.6, (-0.7, -0.4)]. The unit decrease in HbA1c testing frequency among insured individuals was observed across all models, but at a lower level of statistical significance when considering clinical factors and diabetes knowledge and self-care (Models 6 and 7, respectively).

After accounting for SES and living environment, the associations between sex, race/ethnicity, and insurance status remained the same. After consideration of healthcare access and utilization, however, a one-year increase in age was significantly associated with a 0.01 unit decrease in HbA1c testing [-0.01 (-0.01, -0.007)]; age was not previously associated with HbA1c testing in Model 1. All the SES variables were associated with an increase in HbA1c testing, ranging from a 0.02 increase for every dollar increase in income to a 0.3 unit increase in testing for employment status.

We did not observe any significant associations between living environment and HbA1c testing frequency. Having poor mental health was associated with a 0.2-unit increase [0.2 (0.1, 0.3)], and functional independence was associated with a 0.2 unit decrease in HbA1c testing frequency after considering demographic, SES, living environment, and healthcare access and utilization variables [-0.2 (-0.3, -0.1)]. The association between mental health was not significant after consideration of clinical factors and diabetes knowledge and self-care variables. The association between functional independence and HbA1c testing remained significant in Models 5 and 7. The association between depression and HbA1c testing frequency was not significant. Similarly, there was no association between HbA1c testing and duration of diabetes, diabetic eye checks, and physical exercise.

Compared to not using insulin, the use of insulin was associated with a 0.8 unit decrease in HbA1c testing frequency [-0.8 (-0.9, -0.7)]. Having comorbidity was associated with a 0.2 unit increase in HbA1c testing frequency [0.2 (0.1, 0.3)]. Associations between insulin use and comorbidities remained the same after accounting for diabetes knowledge and self-care. Participation in diabetes education was associated with a 0.3 unit decrease in HbA1c testing frequency [-0.3 (-0.4, -0.2)]. Regular blood sugar monitoring was associated with a 0.6 unit decrease in HbA1c testing frequency [-0.6 (-0.8, -0.5)]. Visiting the doctor regularly for a diabetes-related visit was associated with a 1.2 unit increase in HbA1c testing frequency [1.2 (1.05, 1.33)]. Regular foot checks were also associated with a 0.5 unit increase in testing frequency [0.5 (0.4, 0.7)]. After adjusting for all known covariates, age, race, insurance, income, employment status, access to healthcare, clinical factors, diabetic education, regular feet check, and blood sugar monitoring remained significantly associated with the frequency of HbA1c testing.

## Discussion

The present cross-sectional study examined the association between both modifiable and nonmodifiable factors and the frequency of HbA1c testing, using hierarchical multiple linear regression models to examine the association between annual HbA1c testing frequency and demographic, psychosocial, and healthcare access and utilization factors. After adjustment for the various demographic, socioeconomic, healthcare access/ utilization, psychosocial, clinical, and diabetic knowledge factors, the following variables were associated with HbA1c testing frequency: age, race, income, employment status, healthcare access (access to personal doctor and routine checkups), clinical factors (insulin use and comorbidities), and diabetes self-care and knowledge (education, doctor visits for diabetes, foot checks, blood sugar monitoring). Since some of these factors are modifiable, the results of the study could help identify target areas for future intervention to increase HbA1c testing and assure compliance with current diabetes treatment guidelines.

Being American Indians and Alaskans was associated with a higher frequency of annual HbA1c testing. However, the interpretation of this result should be cautious since American Indians and Alaskans comprised only 2% of the total sample. Similarly, the effect of Asian-American ethnicity remains tentative due to the small sample size of this group.

Potentially modifiable factors associated with HbA1c testing frequency included income, education level, healthcare access, and diabetes self-care knowledge. Having a higher income compared to a lower income was associated with an increased frequency of annual HbA1C testing. This is consistent with observations made by Agardh et al., in which individuals with a lower socioeconomic status had worse coping mechanisms in response to adverse events related to their diabetes than those of higher socioeconomic status [14]. Higher levels of education were associated with more frequent HbA1c testing. However, this association was not significant in the final model. Prior studies on this relationship have suggested that individuals with more education have increased awareness of the various complications associated with diabetes. Furthermore, higher levels of education were previously shown to be associated with better adherence to diet and glycemic control [15]. Thus, it appears that higher levels of motivation to adhere to recommendations and guidelines among highly educated and higher earning individuals may contribute to behaviors that contribute to improved outcomes.

Healthcare access is a potentially modifiable factor available for intervention. Having access to a personal physician (e.g., a primary care provider) and more routine check-ups were associated with less frequent HbA1c testing. This finding is surprising, considering that other studies have shown compliance with treatment and follow-up in patients is improved among those who have better access to healthcare [16]. Routine checkups unrelated to diabetes were not associated with an increased or decreased HbA1c testing frequency, but doctor visits for diabetes were associated with an increased HbA1c testing frequency. This finding would be an interesting area for future studies that focus on the role of comprehensive diabetes-specific care.

Comorbid psychiatric disorders in patients with diabetes such as depression can significantly impact the frequency of HbA1c testing as well as general management of blood sugar control. Hence, it is important for primary care physicians and endocrinologists to routinely screen for depression in the clinical setting. This can be fairly easily done by utilizing readily available screening tools such as the PHQ-2 or PHQ-9.

Limitations

There were several limitations to the present study. First, all of the data was self-reported using a phone survey. The sample also had small representations of AI/Alaskan and Asian populations. BRFSS attempts to oversample people who are typically underrepresented in clinical studies; thus, this analysis may have overestimated the effect of minority race/ ethnicity on the frequency of HbA1C testing. Second, 91% of the cohort was insured, which makes it difficult to conclude that the significant association we observed between insurance status and HbA1c testing frequency was in fact significant. Third, another limitation was the lack of temporal data on HbA1C testing frequency. The data points only provide the number of times each respondent tested their HbA1C in a year. It is possible for someone who reported having five HbA1c tests done in a year to have all their testing clustered within the last three months with no testing done during the remaining nine months of the year. Another participant with the same number of HbA1c tests done in a calendar year might have had their tests spread out over a span of a year. It would therefore be essential to collect more detailed information about whether the consistency of regular testing is key to improved diabetes outcomes or whether frequency alone can be beneficial. Fourth, the unit differences we observed were in most cases small (<1.0). Future studies might consider exploring what the minimum change warranting clinical intervention or attention is for HbA1c testing. Future studies on either type I or type II diabetes and HbA1c testing frequency would also be interesting, as testing guidelines are different for each diabetes type.

Strengths

Strengths of the study include the use of a large public, nationally representative data set. The BRFSS data set has been validated and used by several research groups. However, it is important to acknowledge that the BRFSS does not collect diabetes-related variables exclusively. Future studies might use data sets that specifically focus on data related to diabetes, or data sets that include more detailed data that is more relevant to diabetes specifically.

## Conclusions

In summary, this study analyzed the association between annual HbA1c testing frequency and demographic, psychosocial, and healthcare access and utilization factors. There were significant associations between seeing a provider for diabetes, higher levels of income, and higher levels of education with annual HbA1c testing frequency. Future studies might, therefore, examine the effect of a comprehensive diabetes management program that focuses on providing diabetes-specific care. Our data suggest that such a program might be beneficial, especially for those with lower income and education levels.
